# Teaching transposon classification as a means to crowd source the curation of repeat annotation – a tardigrade perspective

**DOI:** 10.1186/s13100-024-00319-8

**Published:** 2024-05-06

**Authors:** Valentina Peona, Jacopo Martelossi, Dareen Almojil, Julia Bocharkina, Ioana Brännström, Max Brown, Alice Cang, Tomàs Carrasco-Valenzuela, Jon DeVries, Meredith Doellman, Daniel Elsner, Pamela Espíndola-Hernández, Guillermo Friis Montoya, Bence Gaspar, Danijela Zagorski, Paweł Hałakuc, Beti Ivanovska, Christopher Laumer, Robert Lehmann, Ljudevit Luka Boštjančić, Rahia Mashoodh, Sofia Mazzoleni, Alice Mouton, Maria Anna Nilsson, Yifan Pei, Giacomo Potente, Panagiotis Provataris, José Ramón Pardos-Blas, Ravindra Raut, Tomasa Sbaffi, Florian Schwarz, Jessica Stapley, Lewis Stevens, Nusrat Sultana, Radka Symonova, Mohadeseh S. Tahami, Alice Urzì, Heidi Yang, Abdullah Yusuf, Carlo Pecoraro, Alexander Suh

**Affiliations:** 1 Department of Organismal Biology – Systematic Biology, Evolutionary Biology Centre, Uppsala University, Uppsala, SE-752 36 Sweden; 2 Swiss Ornithological Institute Vogelwarte, Sempach, CH-6204 Switzerland; 3 Department of Bioinformatics and Genetics, Swedish Natural History Museum, Stockholm, Sweden; 4 Department of Biological Geological and Environmental Science, University of Bologna, Via Selmi 3, Bologna, 40126 Italy; 5 New York University Abu Dhabi, Saadiyat Island, United Arab Emirates; 6 Skolkovo Institute of Science and Technology, Moscow, Russia; 7 Natural History Museum, Oslo University, Oslo, Norway; 8 Department of Ecology and Genetics, Uppsala University, Uppsala, Sweden; 9 Anglia Ruskin University, East Rd, Cambridge, CB1 1PT UK; 10 University of Arizona, Tucson, AZ USA; 11 Evolutionary Genetics Department, Leibniz Institute for Zoo and Wildlife Research, 10315 Berlin, Germany; 12 Berlin Center for Genomics in Biodiversity Research, 14195 Berlin, Germany; 13 Reed College, Portland, OR United States of America; 14 Department of Ecology and Evolution, The University of Chicago, Chicago, IL 60637 USA; 15 Department of Biological Sciences, University of Notre Dame, Notre Dame, IN 46556 USA; 16 Evolutionary Biology & Ecology, University of Freiburg, Freiburg, Germany; 17 Research Unit Comparative Microbiome Analysis (COMI), Helmholtz Zentrum München, Ingolstädter Landstraße 1, D-85764 Neuherberg, Germany; 18 Royal Botanic Gardens, Kew, Richmond, Surrey TW9 3AE UK; 19 Institute of Evolution and Ecology, University of Tuebingen, Tuebingen, Germany; 20 Institute of Botany, Czech Academy of Sciences, Průhonice, Czech Republic; 21 Institute of Evolutionary Biology, Faculty of Biology, Biological and Chemical Research Centre, University of Warsaw, Warsaw, Poland; 22 Institute of Genetics and Biotechnology, Hungarian University of Agriculture and Life Sciences, Budapest, Hungary; 23 The Natural History Museum, Cromwell Road, London, SW6 7SJ UK; 24 Biological and Environmental Science and Engineering Division, King Abdullah University of Science and Technology (KAUST), Thuwal, Saudi Arabia; 25 LOEWE Centre for Translational Biodiversity Genomics (LOEWE-TBG), Senckenberganlage 25, 60325 Frankfurt, Germany; 26 Department of Genetics, Environment & Evolution, Centre for Biodiversity & Environment Research, University College London, London, UK; 27 Department of Ecology, Faculty of Science, Charles University, Prague, Czech Republic; 28 INBIOS-Conservation Genetic Lab, University of Liege, Liege, Belgium; 29 Centre for Molecular Biodiversity Research, Leibniz Institute for the Analysis of Biodiversity Change, Adenauerallee 127, 53113 Bonn, Germany; 30 Department of Systematic and Evolutionary Botany, University of Zurich, Zurich, Switzerland; 31 German Cancer Research Center, NGS Core Facility, DKFZ-ZMBH Alliance, 69120 Heidelberg, Germany; 32 Departamento de Biodiversidad y Biología Evolutiva, Museo Nacional de Ciencias Naturales (MNCN-CSIC), José Gutiérrez Abascal 2, Madrid, 28006 Spain; 33 Department of Biotechnology, National Institute of Technology Durgapur, Durgapur, India; 34 Molecular Ecology Group (MEG), National Research Council of Italy – Water Research Institute (CNR-IRSA), Verbania, Italy; 35 Eurofins Genomics Europe Pharma and Diagnostics Products & Services Sales GmbH, Ebersberg, Germany; 36 Plant Pathology Group, Institute of Integrative Biology, ETH Zurich, Zurich, Switzerland; 37 Tree of Life, Wellcome Sanger Institute, Cambridge, CB10 1SA UK; 38 Department of Botany, Jagannath Univerity, Dhaka, 1100 Bangladesh; 39 Institute of Hydrobiology, Biology Centre of the Czech Academy of Sciences, České Budějovice, Czech Republic; 40 Department of Biological and Environmental Science, University of Jyväskylä, P.O. Box 35, Jyväskylä, 40014 Finland; 41 Centogene GmbH, Am Strande 7, 18055 Rostock, Germany; 42 Department of Ecology & Evolutionary Biology, University of California, Los Angeles, Los Angeles, CA United States of America; 43 Zell- und Molekularbiologie der Pflanzen, Technische Universität Dresden, Dresden, Germany; 44 Physalia-courses, 10249 Berlin, Germany; 45 School of Biological Sciences, University of East Anglia, Norwich Research Park, Norwich, NR4 7TU UK; 46 Present address: Centre for Molecular Biodiversity Research, Leibniz Institute for the Analysis of Biodiversity Change, Adenauerallee 160, 53113 Bonn, Germany

**Keywords:** Transposable elements, Manual curation, Library, Annotation, Non-model organism, Genome assembly

## Abstract

**Background:**

The advancement of sequencing technologies results in the rapid release of hundreds of new genome assemblies a year providing unprecedented resources for the study of genome evolution. Within this context, the significance of in-depth analyses of repetitive elements, transposable elements (TEs) in particular, is increasingly recognized in understanding genome evolution. Despite the plethora of available bioinformatic tools for identifying and annotating TEs, the phylogenetic distance of the target species from a curated and classified database of repetitive element sequences constrains any automated annotation effort. Moreover, manual curation of raw repeat libraries is deemed essential due to the frequent incompleteness of automatically generated consensus sequences.

**Results:**

Here, we present an example of a crowd-sourcing effort aimed at curating and annotating TE libraries of two non-model species built around a collaborative, peer-reviewed teaching process. Manual curation and classification are time-consuming processes that offer limited short-term academic rewards and are typically confined to a few research groups where methods are taught through hands-on experience. Crowd-sourcing efforts could therefore offer a significant opportunity to bridge the gap between learning the methods of curation effectively and empowering the scientific community with high-quality, reusable repeat libraries.

**Conclusions:**

The collaborative manual curation of TEs from two tardigrade species, for which there were no TE libraries available, resulted in the successful characterization of hundreds of new and diverse TEs in a reasonable time frame. Our crowd-sourcing setting can be used as a teaching reference guide for similar projects: A hidden treasure awaits discovery within non-model organisms.

**Supplementary Information:**

The online version contains supplementary material available at 10.1186/s13100-024-00319-8.

## Background

The importance of in-depth analyses of repetitive elements, particularly transposable elements (TEs), is becoming more and more fundamental to understand genome evolution and the genetic basis of adaptation [1]. While there is a wealth of bioinformatic tools available for the identification and annotation of TEs (https://tehub.org/en/resources/repeat_tools), any automated annotation effort is limited by the phylogenetic distance of the target species to a database of curated and classified repetitive element sequences [2]. For example, in birds where zebra finch and chicken have well-characterized repetitive elements because their genomes were first sequenced in large consortia during the pre-genomics era [3, 4], automated annotation of other bird genomes will render most repeats as correctly classified [5, 6]. On the other hand, in taxa as diverse and divergent as insects, up to 85% of repetitive sequences can remain of “unknown” classification in non-*Drosophila* species [7]. This is problematic. Inferences about the mobility and accumulation of TEs, as well as their potential effects on the host, are not feasible for unclassified repeats, as well as for incorrectly classified repeats if the automated classification is based on short, spurious nucleotide sequence similarity [8, 9].

The reference bias in TE classification reflects the history of the TE field in the genomics era: In the 1990s and 2000s, there were usually multiple people tasked with TE identification, classification, and annotation for each genome project, yielding manually curated TE consensus sequences (namely representative sequences whose quality was manually controlled and improved) and fully classified TE libraries deposited in databases such as Repbase [2]. Over the last ten years, however, the number of genome projects both of individual labs as well as large consortia has increased exponentially and so have speed and number of automated TE annotation efforts [10–12], while time and personnel have remained limited for curated TE annotation efforts. Similar to taxonomic expertise required for identifying and classifying organisms, TE identification and classification need hands-on experience with manual curation for months or even years per genome [1] which is usually taught through knowledge passed within genome projects and research groups. Recent efforts [13–15] have started to make manual curation accessible to a broader scientific audience, with the aim to increase reproducibility and comparability. However, what cannot be changed is that there are hundreds if not thousands of genomes per TE-interested researcher with more or less pressing priority for time-consuming manual curation.

Low scalability and people power are major obstacles that need to be overcome by the many facets of computational biology where curation is essential. Annotation efforts of other genomic features have shown that crowd sourcing through teaching [16–22], or “course sourcing” as we call it, has the benefit of providing participants with hands-on skills for curation and experience on how to reconcile biology with technical limitations, while simultaneously sharing the workload of time-consuming curation across multiple people working on different parts at the same time. Thus, we argue that a TE curation effort that would take months or years for a single person may fit into a few days or weeks of teaching, of course as long as reproducibility and comparability are ensured throughout course duration.

Here, we present our “course sourcing” experience from two iterations of a Physalia Course on TE identification, classification, and annotation. We focused on two species of tardigrades as a case study to motivate student-centered learning through direct contribution to scientific knowledge: Tardigrades are, to our knowledge, the most high-ranking animal phylum without curated TE annotation, very clearly illustrated by the fact that in previous genome analyses, almost all repeats remained of “unknown” classification [23]. Tardigrades are a diverse group of aquatic and terrestrial animals which show extraordinary ability to survive extreme environments by entering the state of cryptobiosis [24]. This animal clade comprises almost 1,200 described species belonging to Panarthropoda [25] and the two species used in the courses are closely related and belong to the Hypsibiidae family [23].

The first course took place in person in June 2018 in Berlin across five full-time work days: The first three days familiarized the 13 participants with the biology of TEs, concepts for classification, and methods for annotation using the tardigrade *Hypsibius exemplaris* genome (formerly identified as *Hypsibius dujardini*), while the last two days had a student-centered learning format where each participant was able to curate as many TEs as possible from the target species. The second course took place virtually in June 2021 due to the Covid-19 pandemic and comprised five afternoons in the Berlin time zone to minimize Zoom fatigue. The overall format was similar to the prior in-person course but with 24 participants and focusing on another tardigrade, *Ramazottius varieornatus*, which the participants identified to have not a single shared TE family with the tardigrade *H. exemplaris* curated in the 2018 course. Between the two courses, the participants were able to uncover a vast diversity of TEs and successfully curate over 400 consensus sequences. We demonstrate therefore that a collaborative approach is a valuable means to achieve significant results for the scientific community and we hope to share with the community a teaching reference for future similar efforts, because: A hidden treasure always awaits discovery in non-model organisms.

## Results and discussion

**Fig. 1:**
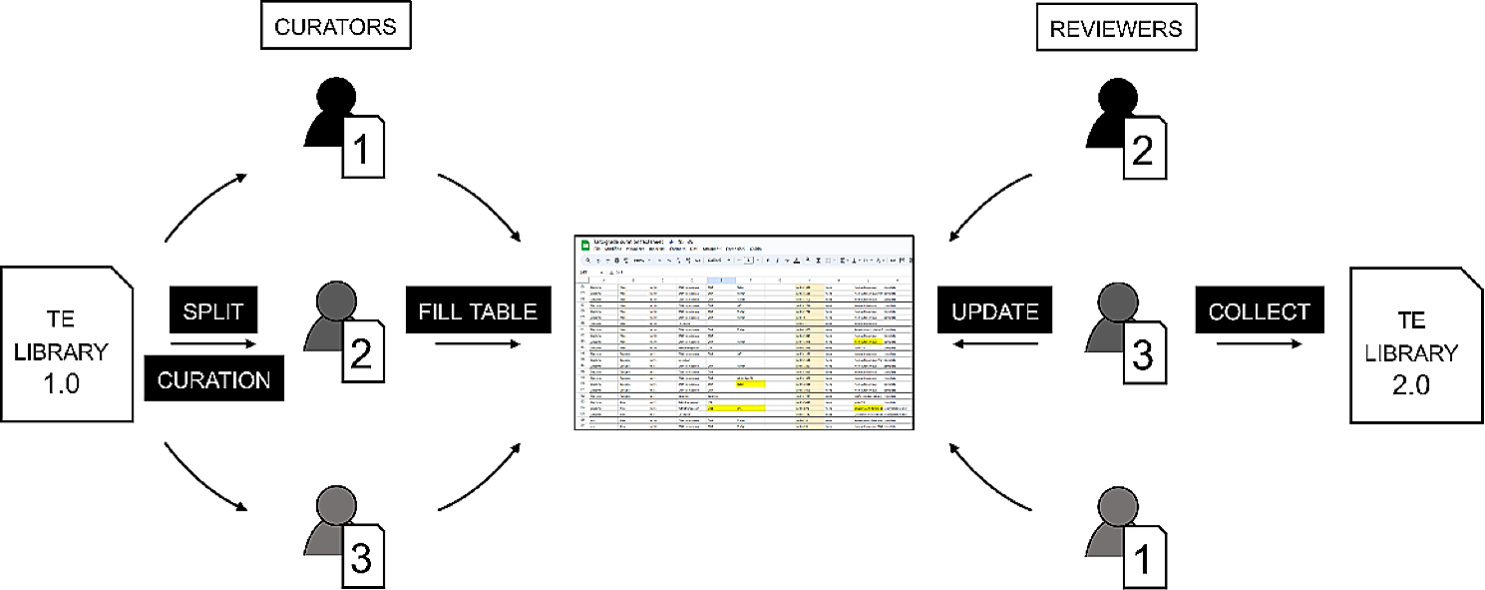
Schematic representation of the peer-reviewed process of TE curation

Incorporating crowd sourcing efforts within a classroom setting (“course sourcing”) can represent an invaluable opportunity for teaching, while simultaneously contributing to the scientific community. However, course sourcing also does present its own unique challenges, particularly in terms of minimizing errors, maximizing reproducibility and student engagement. Drawing from our experience in both in-person and virtual settings, we identified several crucial factors in teaching TE manual curation that must be considered during the organization and supervision of such courses, like: (a) establishing a standardized approach for curation and classification of TE consensus sequences; (b) implementing a peer-review process between participants to check on the quality of the curation of each TE consensus sequence; (c) maintaining meticulous version control of the libraries. Here, we describe how we addressed these points. First, to establish a standard approach to manual curation, we implemented methods widely used in the TE community that have been recently reviewed in detail [13, 14]. The approach, briefly, consists in producing and inspecting multi-sequence alignments for each of the consensus sequences automatically generated by RepeatModeler [10]. Each nucleotide position of the “alignable part” of the alignment is carefully inspected to identify the correct termini of the TE while correcting for any ambiguous base or gap. To correct for ambiguous bases in the curated consensus sequence, we applied the majority rule and assigned the most representative IUPAC nucleotide character for each position in the alignment (see Methods). To correct the consensus sequences where gaps of different lengths are present, we considered each insertion/deletion length as independent events so that a majority rule was applicable to these regions as well. When very complex regions could not be unambiguously solved, stretches of 10 N nucleotides were inserted as placeholder (gap) in the consensus sequence. The TE classification followed the nomenclature used by RepeatMasker to ensure direct compatibility with the tool and its suite of scripts for downstream analysis. Second, when participants completed the curation of their consensus sequences, then their results would go through a peer-review process where both the quality of each consensus sequence and its classification were revised by other participants (or course faculty). During the in-person edition, a random set of consensus sequences curated by one participant was assigned to another participant, while in the second online edition, all sequences were reviewed by the two instructors and one participant (Fig. 1). The review of the TE sequences continued after the official conclusion of the course. To ensure reproducibility and the documentation of the entire decision-making process for classification, all steps and details of classification were recorded in a shared Google Sheet. The tables would include the changes in consensus sequence names, names of the curators and reviewers as well as additional comments (Fig. 1, Table S1). Whenever a change was introduced in a consensus sequence (either in the nucleotide sequence itself or in the classification), the new version was directly added to the multi-sequence alignment file used for curation together with the original one. Keeping all the versions of a consensus in the same alignment file and respective notes in the tables allows the implementation of a basic version control useful to check on the steps leading to a particular decision. From the re-iteration of the course, we noticed three particularly challenging points for beginners that need an extra supervision effort. The most challenging points are the identification of the correct termini, target site duplications (a hallmark of transposition for the vast majority of TEs) if any, and the correct spelling of the TE categories for classification in accordance with the RepeatMasker nomenclature rules. The last point is of particular importance especially if the repeat annotation is visualized as a landscape using the RepeatMasker scripts (e.g., calcDivergenceFromAlign.pl and createRepeatLandscape.pl) to avoid causing computing errors and downstream misinterpretations.

Finally, all the tutorials to obtain and curate a TE library are available on the GitHub repository linked to this paper: https://github.com/ValentinaPeona/TardigraTE.

### Improvement of the transposable element libraries

To generate the TE libraries, we first ran RepeatModeler and RepeatModeler2 on *H. exemplaris* and *R. varieornatus*, respectively, and obtained 519 and 898 consensus sequences (Table 1). Then the course participants manually curated as many consensus sequences as possible. In about three course days plus voluntary efforts by some participants after each course, the participants were able to curate 274 consensus sequences (53%) of the *H. exemplaris* library and 139 consensus sequences (15%) of the *R. varieornatus* library (Table S1-3). Given the lack of previously curated libraries from closely related species, most of the consensus sequences were automatically classified as “Unknown” by RepeatModeler, but the thorough process of manual curation successfully reclassified 296 unknown consensus sequences (out of a total of 413 curated sequences, 71%) into known categories of elements. After manual curation, we found that most of the two species’ libraries are comprised of DNA transposons and a minority of retrotransposons (Table 1). Since many consensus sequences remained uncurated and unclassified, it is possible that the relative percentages of the categories change in the future, but we expect, especially from the composition of the *H. exemplaris* library, to mostly find additional (non-autonomous) DNA transposons among the unclassified.

**Table 1:** Overview of classification of tardigrade repeats in the curated libraries. The libraries here described contain both curated and uncurated consensus sequences. The number of automatically classified elements in the original raw libraries are reported in parentheses

Species	DNA	LINE	LTR	SINE	Unknown
***Hypsibius exemplaris***	237 (41)	12 (12)	29 (8)	2 (2)	199 (394)
***Ramazzottius varieornatus***	200 (89)	35 (35)	11 (10)	- (1)	651 (758)

**Fig. 2:**
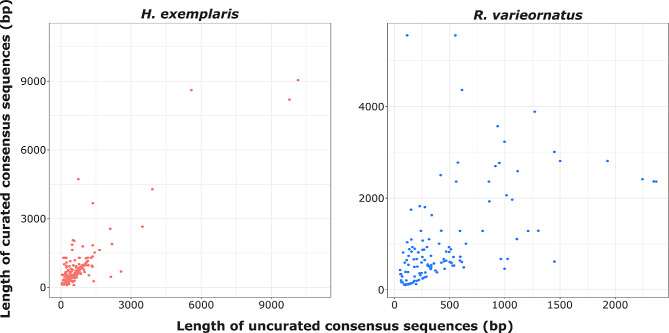
Comparison of the length of the consensus sequences before and after manual curation

The process of manual curation improved the overall level of TE classification of the libraries but also the quality of the individual consensus sequences by correctly identifying their termini and in general by extending their sequence. Indeed, by comparing the lengths of the consensus sequences for the same element, we can notice a marked increase in length after curation (Fig. 2).

### Diversity of transposable elements

**Fig. 3:**
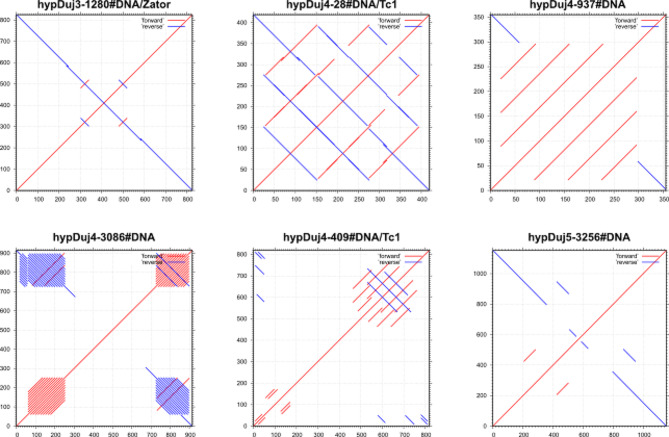
Dotplots of six DNA transposons from the library of *Hypsibius exemplaris* produced with the MAFFT online server. These elements were selected by course participants for aesthetic reasons

When looking at the diversity of repeats in the curated libraries (combined libraries comprising curated and uncurated consensus sequences), we identified a total of 437 Class II DNA consensus sequences belonging to the superfamilies/clades CMC, MULE, TcMar, Sola, PiggyBac, PIF-Harbinger, Zator, hAT, Maverick, P and Zisupton. Many of these elements are non-autonomous and show a remarkable diversity and complexity of internal structures (Fig. 3) which emphasizes the need for complete, curated consensus sequences to be able to properly classify internal repeat structures and infer their mode of accumulation in the genome. For Class I retrotransposons, we found 47 LINEs belonging to the superfamilies/clades L1, I, CR1, CRE, R2, R2-NesL, L2, RTE-X and RTE-BovB and another 40 LTRs belonging to the superfamilies/clades DIRS, Gypsy, Ngaro and Pao. The REPET library generated for *R. varieornatus* (Table S4) consists of a total of 130 consensus sequences, with the majority classified as DNA transposons (129), similar to the curated consensus generated from RepeatModeler output (Table S3). However, several superfamilies manually identified in the RepeatModeler library, including Zator, MULE, and P, were not detected in the REPET one. These differences may be attributed to variations in underlying software and also to differences in curation and decision-making processes.

**Fig. 4:**
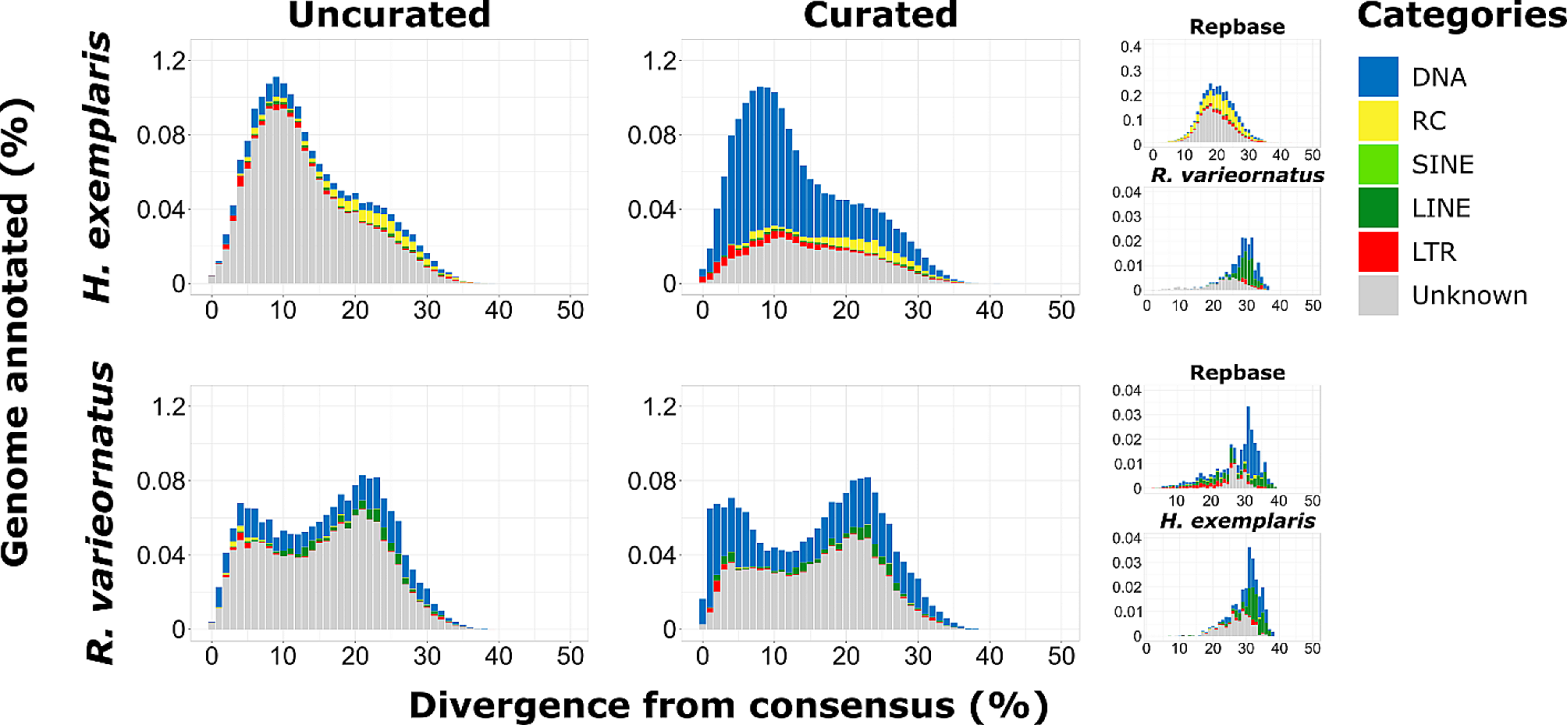
Repeat landscapes of the genomes of *H. exemplaris* and *R. varieornatus* annotated with the Repbase (Arthropoda clade), uncurated and curated of both tardigrades combined libraries, and with libraries of the reciprocal species (only species-specific repeats). The divergence from consensus calculated with the Kimura 2-parameter distance model is shown on the x-axis. The percentage of genome annotated is shown on the y-axis

To highlight the importance of generating and using custom repeat libraries for the organisms of interest as well as their curation, we masked the two tardigrade genomes and compared how the annotation and accumulation patterns change when using general repeat libraries (in this case the Repbase library for Arthropoda) and species-specific ones before and after curation (Fig. 4; Table 2 and S5). The use of the known repeats for Arthropoda available on Repbase provided a poor and insufficient annotation for both species (all the following percentages are given for *H. exemplaris* and then for *R. varieornatus*) where only 1.95% and 0.26% of the assemblies were annotated as interspersed repeats and the accumulation patterns were characterized only by likely old insertions. Then the use of species-specific, albeit uncurated, libraries completely changed the percentage of TEs annotated (16.38% and 15.66%) and their accumulation patterns that showed many recently accumulated insertions. While the shape and percentages of the repeat landscapes did not drastically change after the manual curation of the libraries, the curated libraries clearly highlighted a large accumulation of DNA transposons in recent and ancient times alike that were either not present in the other landscapes or were hidden among the “unknown” repeats. Especially for *R. varieornatus*, the curation highlighted a higher accumulation of repeats in the very recent times (1–5% of divergence). This higher accumulation of DNA transposons in recent times is also in line with the finding of multiple putatively active transposable element subfamilies (Table 3). Finally, the use of the repeat library of one species to annotate the other species (reciprocal masking) resulted to be almost as insufficient as the use of the Repbase library for Arthropoda, stressing once again how important it is to have a capillary knowledge of the repeatome for correct biological interpretations.

**Table 2:** Number of base pairs annotated and percentages of the main TE categories. The full version of this table with information about the annotation with Repbase Arthropoda and reciprocal libraries can be found in Table S5

Species	Library	DNA (bp)	DNA (%)	LINE (bp)	LINE (%)	SINE (bp)	SINE (%)	LTR (bp)	LTR (%)	Unknown (bp)	Unknown (%)	Total (bp)	Total (%)
*Hypsibius exemplaris*	Uncurated	1,681,052	1.65	310,239	0.3	5166	0.01	514,564	0.5	14,199,202	13.92	16,710,223	16.38
	Curated	11,149,552	10.93	290,632	0.28	2424	0	868,156	0.85	4,658,887	4.57	16,969,651	16.63
*Ramazzottius varieornatus*	Uncurated	1,753,754	3.16	413,647	0.75	4486	0.01	134,451	0.24	6,375,274	11.5	8,681,612	15.66
	Curated	3,385,077	6.11	454,742	0.82	1320	0	145,257	0.26	4,880,857	8.81	8,867,253	16

**Table 3:** List of repeat subfamilies with putatively ongoing activity, i.e., at least 10 copies with 0% distance to consensus

TE category	Hypsibius exemplaris	Ramazzottius varieornatus
DNA transposon	7	3
LTR retrotransposon	3	0
Unknown	0	2

**Fig. 5:**
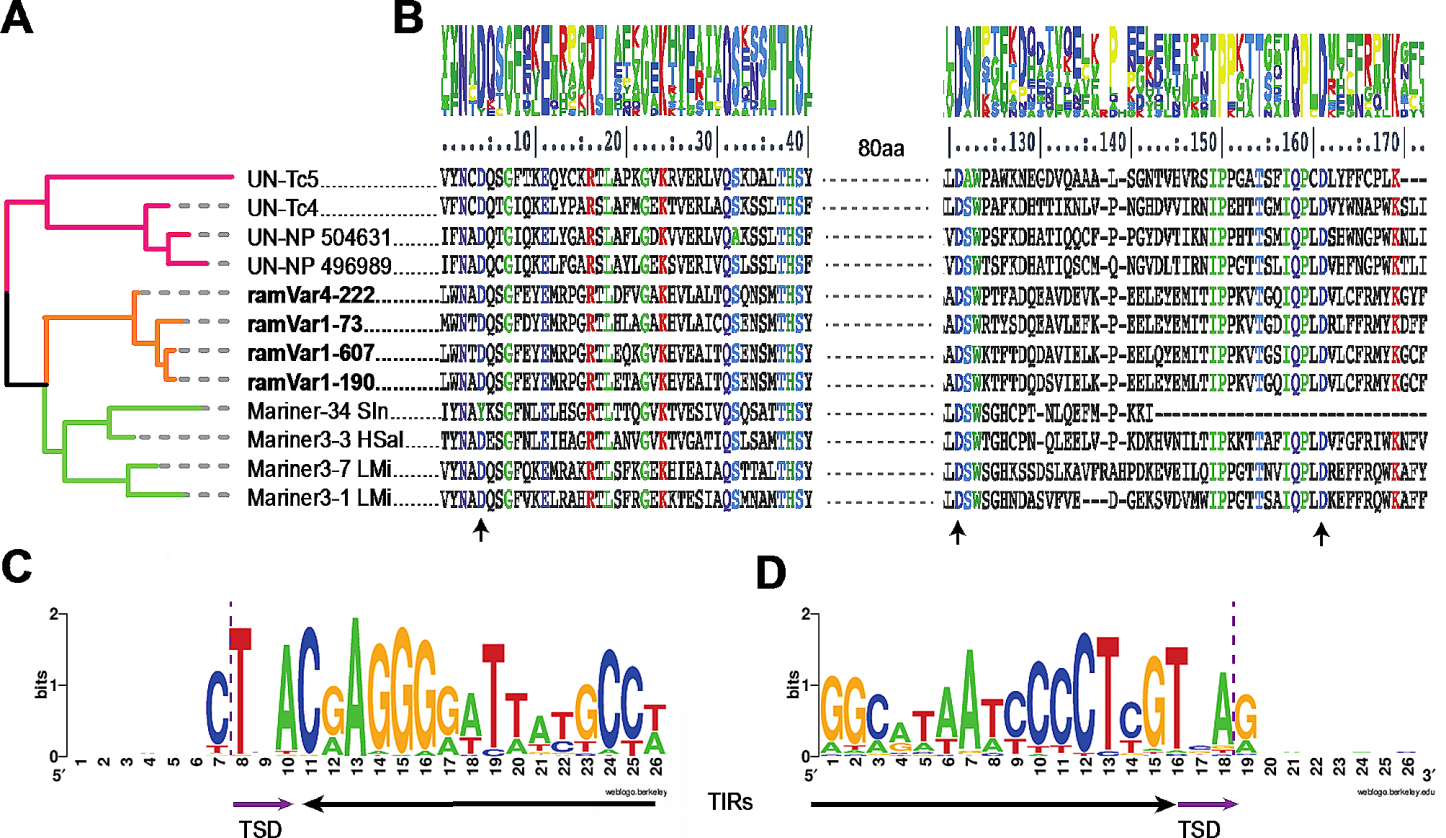
Characterization and phylogenetic analyses of Tc4 elements. (**A**) Phylogenetic tree of Tc4 consensus sequences based on DDD catalytic domains identified in the *R. varieornatus* consensus sequences, highlighted in bold and orange, together with representative sequences extracted from the RepeatPeps library from nematodes (pink) and insects (green). All nodes received maximal support value. (**B**) Alignment of DDD catalytic domains of sequences included in phylogenetic analyses. Residues conserved in more than 80% of the sequences are colored. Arrows highlight catalytic DDD residues. Sequence logos of 5’ (**C**) and 3’ (**D**) ends of Tc4 elements used to curate the *R. varieornatus* consensus sequences. Black and purple arrows denote terminal inverted repeats (TIRs) and target site duplications (TSDs), respectively. The purple dotted line marks the transposase cut on the CTNAG target site

As a demonstrative example of the contribution of the collaborative curation process in providing novel insights into TE diversity, taxonomic distribution and biology, we decided to deeply characterize consensus sequences that we classified as Tc4. These elements have a rather limited taxonomic distribution, few references in the literature exist, and they incompletely duplicate the target site upon transposition [26] which can impose challenges for their classification. The Tc4 transposons are DDD elements firstly discovered in *Caenorhabditis elegans* [26] where they recognize the interrupted palindrome CTNAG as target site for insertion, and cause duplication of only the central TNA trinucleotide. Regarding their taxonomic distribution, consensus sequences for Tc4 elements are known and deposited only for nematodes and arthropods in RepeatPeps, Repbase and DFAM. Phylogenetic analyses based on DDD segments confidently placed the four tardigrade Tc4 consensus sequences identified in *R. varieornatus* within the Tc4 clade in a sister relationship with arthropod elements and with a branching pattern that reassembles the Panarthropoda group (tardigrades + onychophorans + arthropods) within Ecdysozoa [27] (Fig. 5A). The DDD catalytic domain is highly conserved between different phyla (Fig. 5B) and the target site of tardigrades mirrors what was previously observed in nematodes (i.e., C|TNA|G where “|” marks the transposase cut site; Fig. 5C-D). We could therefore hypothesize that these elements first originated during the diversification of Ecdysozoa. However, broader comparative analyses involving more early-diverging Metazoa clades are necessary to confirm this lineage-specific origin.

### Contributions from the course participants

During both editions of the course, participants were free to explore their favorite topics within the scope of the syllabus and we share two contributions developed by the participants that can be useful for the entire community. First, an additional repeat library of 130 consensus sequences (119 of which are DNA transposons) was produced with the use of REPET for *R. varieornatus* (Table S4). Second, a guide for the classification of TEs from multisequence alignments (File S1) that can be a useful starting point for beginners and complementary to more extensive guides [13, 14].

## Conclusion

As shown here and in many other studies, repeat annotation is key to correctly identify and interpret patterns of genome evolution and proper annotation is based on a thorough curation of the repeat libraries [8, 9, 28]. However, it is hard for curation efforts to keep up with the sheer number of genome assemblies released every year as curation done by single laboratories may require months or even years for a single genome. Tools like TE-Aid [13] and EarlGrey [29] are rapidly spreading and gaining popularity to facilitate TE curation processes [30–33]. Despite these advancements, until fully automatized, reliable tools are developed and there are manual curation training sets for understudied taxa, we emphasize the need to implement manual curation for repeat libraries as well as to find alternative ways to deal with the curation of hundreds of new libraries. Here we presented one such alternative approach, namely a peer-reviewed course sourcing effort designed to be as reproducible and comparable as possible and where the hands-on tutorials were designed to be meaningful for the participants because they dealt with real unexplored data and directly contributed to the scientific community. The two iterations of this course sourcing effort resulted in the successful curation of hundreds of new and diverse TEs. While the repeat libraries presented here were not completely curated and classified, we would like to highlight that TE curation can be considered as a “cumulative” effort of a community. The more people learn how to curate, the more teachers are educated and the faster the process becomes. Therefore, we hope that this experience and teaching framework can be of use for the genome research community and that it can be applicable to other types of data/analyses that need manual curation (e.g., genome assemblies [21, 22] and gene annotations).

## Materials and methods

### Genome assemblies

For this study, we used the genome assemblies of the two tardigrade species: *Hypsibius exemplaris* (formerly identified as *Hypsibius dujardini*; GCA_002082055.1) and *Ramazzottius varieornatus* (GCA_001949185.1) produced by sequencing a pool of male and female individuals by Yoshida et al. [23]. The *Hypsibius exemplaris* genome was assembled using long PacBio and short Illumina reads whereas the *Ramazzottius varieornatus* genome was assembled using a combination of Sanger and Illumina reads [23].

### Raw repetitive element library

To start the *de novo* characterization of TEs, we ran RepeatModeler on *H. exemplaris* and RepeatModeler2 on *R. varieornatus* [34] using the option -LTR_struct and obtained a library of raw consensus sequences for each of the genomes. RepeatModeler and not RepeatModeler2 was used on *H. exemplaris* since at the time of the first edition of the course in 2018, only RepeatModeler was available. RepeatModeler and RepeatModeler2 automatically named the consensus sequences with the prefix “rnd” that we replaced with the abbreviations of the species names: “hypDuj” for *H. exemplaris* and “ramVar” for *R. varieornatus*. Note that the abbreviation “hypDuj” was assigned prior to the scientific name change from *H. dujardini* to *H. exemplaris*. Despite this, we have chosen to retain “hypDuj” in the final repeat library for the sake of simplicity.

The two libraries were then compared to find similar sequences belonging either to the same family or subfamily by using, respectively, the 80-80-80 rule [35] and the 95-80-98 rule [36]. The rules were applied by masking the library of *R. varieornatus* with the library of *H. exemplaris* using RepeatMasker [37] and by parsing the resulting. out table with awk.

### Manual curation of the consensus sequences

After the generation of the libraries of raw consensus sequences, we proceeded with the collaborative peer-reviewed manual curation step. For example in the second iteration of the course, the participants were split into ten groups and each group received about 80 consensus sequences to curate.

The curation of the raw consensus sequences followed a “Blast-Extend-Extract” process. The first step of the curation consisted in the alignment of the raw consensus sequences to the genome of origin using BLAST [38]. The best 20 BLASTN hits were selected, extended by 2 kb at both ends and aligned to their raw consensus sequence with MAFFT [39] which produced a multisequence alignment for each consensus sequence ready to be manually curated (script RMDL_curation_pipeline.pl, first published in [40]).

Each of the multisequence alignment was then inspected to: (1) find the actual boundaries of the repetitive element; (2) build a new consensus sequence with Advanced Consensus Maker (https://hcv.lanl.gov/content/sequence/CONSENSUS/AdvConExplain.html); (3) fix ambiguous base and gap calls in the new consensus sequence following the majority rule; (4) find sequence hallmarks to define the repetitive elements as transposable elements (e.g., target site duplication, long terminal repeats, terminal inverted repeats or other motifs). Every new consensus sequence was reported in a common Excel table (Table S1). To quantitatively measure the improvement of the repeat libraries after manual curation, we compared the length of consensus sequences before and after curation.

In all the figures and tables, the term “curated” indicates that the library mentioned contains manually curated consensus sequences as well as all the consensus sequences that remained uncurated. Finally, we consider each consensus sequence as a proxy for a transposable element subfamily. However, the consensus sequences were not checked for redundancy and not clustered into families and subfamilies using the 80-80-80 or 95-80-98 rules for nomenclature because the focus of the study was on classifying the consensus sequences into superfamilies and orders of transposable elements.

The code used to produce the consensus sequences and their alignments is provided as tutorial on the GitHub repository https://github.com/ValentinaPeona/TardigraTE.

### Classification

The new consensus sequences were classified using sequence characteristics retrieved by the alignments (e.g., target site duplications, terminal repeats) and homology information retrieved through masking the sequences with Censor [41, 42] following the recommendations from [35] and [43]. When the information retrieved by the alignments and Censor was not enough to provide a reliable classification of the elements, the sequences were further analyzed for the presence of informative protein domains using the Conserved Domain Database [44–46].

Since the course participants in general had never curated transposable element alignments before, we decided to implement a peer-review process. For the first course (*H. exemplaris*), the results of each participant were sent to another participant to check the curated alignments and independently retrieve key information for the classification. The independent sequences and classifications would be compared and fixed if necessary. In the second course (*R. varieornatus*), all sequences were inspected by the same 3 reviewers and only these applied the same process as previously described.

### Comparative analysis of the repetitive content

The genome assemblies of both tardigrade species were masked with RepeatMasker 4.1.10 using four different types of TE libraries: (1) known Arthropoda consensus sequences from Repbase; (2) uncurated raw consensus sequences from the respective species; (3) curated consensus sequences together with the consensus sequences that were not curated from the respective species; (4) curated consensus sequences together with the consensus sequences that were curated from the other species. The RepeatMasker output files were then used to get the percentages of the genomes annotated as TEs and to visualize the landscapes of the accumulation of repeats.

Finally, we estimated the number of putative active transposable elements in the two genomes by filtering the RepeatMasker annotation for elements that show at least 10 copies with 0% divergence from their consensus sequences.

### Characterization of Tc4 elements

During the manual curation process, participants found types of DNA transposons that are currently considered to have a rather restricted phylogenetic distribution like Tc4 elements, therefore more in-depth analyses were run on these elements. The protein domains of known Tc elements were compared to the Tc4 consensus sequences from the tardigrade species and phylogenetic relationships were established.

Protein homologies of the partially curated repeat libraries were collected using BLASTX (e-value 1e-05) [47] against a database of TE-related protein (RepeatPeps library) provided with the RepeatMasker installation. We extracted the amino acid translation of each hit on Tc4 elements based on the coordinates reported in the BLASTX output. Resulting protein sequences were aligned together with all members of the TcMar group present in RepeatPeps library using MAFFT (*L-INS-i* mode) [48] and the alignment was manually inspected to identify and isolate the catalytic DDD domain. The resulting trimmed alignment was used for phylogenetic inference with IQ-TREE-2 [49], identifying the best-fit evolutionary model with ModelFinder2 and assessing nodal support with 1000 UltraFastBootstrap replicates [50]. The resulting maximum likelihood tree was mid-point rooted and the Tc4 subtree extracted for visualization purposes. The alignment with all members of the TcMar superfamily and the resulting phylogenetic tree can be found in File S2 and S3, respectively.

The DDD segments of Tc4 elements were re-aligned using T-Coffee in *expresso* mode [51] to produce conservation scores. A sequence logo of 5’ and 3’ boundaries of identified Tc4 elements was produced extracting all sequences used to curate the four *R. varieornatus* Tc4 elements and keeping the first 15 bp and 11 bp before and after the terminal inverted repeats (TIRs), respectively.

### Additional transposable element library

Participants ran REPET V3.0 [52] to produce a *de novo* transposable element library for *R. varieornatus* in parallel to the one generated by RepeatModeler2. A custom TE library composed by repeats from Repbase and from *H. exemplaris* was used to aid REPET in the classification process. Only consensus sequences that showed two or more full-length copies in the *R. varieornatus* genome were retained in the new library. Furthermore, the consensus sequences were scanned for protein domains and presence of TIRs or long terminal repeats (LTRs).

### Electronic supplementary material

Below is the link to the electronic supplementary material.

Supplementary Material 1

Supplementary Material 2

Supplementary Material 3

Supplementary Material 4

## Data Availability

Data is provided within the manuscript or supplementary information files.

## Abbreviations

LTR: Long Terminal Repeats
TE: Transposable Element
TIR: Terminal Inverted Repeats
